# Characterisation of Cultured Mesothelial Cells Derived from the Murine Adult Omentum

**DOI:** 10.1371/journal.pone.0158997

**Published:** 2016-07-12

**Authors:** Sumaya Dauleh, Ilaria Santeramo, Claire Fielding, Kelly Ward, Anne Herrmann, Patricia Murray, Bettina Wilm

**Affiliations:** 1 Department of Cellular and Molecular Physiology, Institute of Translational Medicine, University of Liverpool, Liverpool, United Kingdom; 2 Department of Biochemistry, Institute of Integrative Biology, University of Liverpool, Liverpool, United Kingdom; The Roslin Institute, UNITED KINGDOM

## Abstract

The human omentum has been long regarded as a healing patch, used by surgeons for its ability to immunomodulate, repair and vascularise injured tissues. A major component of the omentum are mesothelial cells, which display some of the characteristics of mesenchymal stem/stromal cells. For instance, lineage tracing studies have shown that mesothelial cells give rise to adipocytes and vascular smooth muscle cells, and human and rat mesothelial cells have been shown to differentiate into osteoblast- and adipocyte-like cells *in vitro*, indicating that they have considerable plasticity. However, so far, long-term cultures of mesothelial cells have not been successfully established due to early senescence. Here, we demonstrate that mesothelial cells isolated from the mouse omentum could be cultured for more than 30 passages. While epithelial markers were downregulated over passages in the mesothelial cells, their mesenchymal profile remained unchanged. Early passage mesothelial cells displayed clonogenicitiy, expressed several stem cell markers, and up to passage 5 and 13, respectively, could differentiate along the adipogenic and osteogenic lineages, demonstrating stem/progenitor characteristics and differentiation potential.

## Introduction

The development of regenerative medicine therapies (RMTs) has become a major research focus, with the aim to test and establish approaches that allow repair of damaged tissues and organs. Stem or progenitor cells play prominent roles in this field based on the hypothesis that they can be utilised to contribute to regenerative or repair mechanisms by integrating into the damaged site, replacing lost cells and ameliorating tissue damage as well as loss of functionality. A promising source of RMTs comprises adult resident stem or progenitor cells, which are thought to contribute to the regulation of normal tissue homeostasis [1].

In recent years, findings by several groups have supported the notion that mesothelial cells isolated from adult rodents have regenerative potential [2–5]. Mesothelial cells constitute a simple squamous epithelium that lines the coelomic cavities as parietal mesothelium and surrounds the organs within coelomic cavities as visceral mesothelium. During embryonic development, the mesothelium arises as an epithelium from the mesoderm [6–8], however, mesothelial cells have been shown to express both epithelial and mesenchymal markers [9] (reviewed in [3]). Lineage tracing studies have demonstrated that mesothelial-derived cells contribute to the vasculature of the heart, lung and intestine via epithelial-to-mesenchymal transition (EMT) and differentiation into vascular smooth muscle cells [10–13]. In the adult, mesothelia persist throughout life covering the heart, intestine and associated glands and tissues, the lungs, and the reproductive organs [3, 14]. Generally, the serosal mesothelia contribute to the frictionless movement of the intestinal loops, and have been attributed immune-secretory functions [3]. However, genetic lineage tracing studies have demonstrated that mesothelial-derived cells contribute to the formation of the visceral white adipose tissue during gestation and in the young adult mouse; the mesothelial marker Wilms tumour 1 (Wt1) is required for this process [14, 15]. Furthermore, using mesothelin (Msln)-based lineage tracing, it was shown that mesothelial cells give rise to the majority of visceral smooth muscle and the fibroblast lineage in coelomic organs throughout embryonic development, and maintain tissue homeostasis in the adult [16].

Under pathological conditions, mesothelia respond in a range of ways: repeated exposure to hyperglycemic or bio-incompatible peritoneal dialysis solutions can lead to fibrosis, and in severe cases to encapsulating peritoneal sclerosis of the peritoneal mesothelium [17, 18]. Furthermore, injury to the visceral mesothelium during abdominal surgery can result in adhesions through persistence of fibrin clots, resulting in the formation of fibrous bands [19–21].

These findings indicate that mesothelial cells can undergo trans-differentiation to a fully mesenchymal phenotype, and in pathological conditions, can contribute to fibrosis and sclerosis formation by generating large amounts of extracellular proteins, including collagen. While mesothelial cells have been shown to be free-floating, and contribute to the recovery of de-nuded mesothelial peritoneal membranes [22], approaches to utilise mesothelial cells in peritoneal repair have not been successfully established so far.

Mesothelial cells have been postulated to be involved in the repair processes in the heart after infarction or amputation. Specifically, in experimental animal systems, injury to or amputation of part of the ventricle results in activation of the adult epicardium, leading to the contribution of epicardial cells to the regeneration of the myocardium and cardiovascular system [23–25]. These findings suggest that cells of the epicardium have progenitor properties [26]. Further evidence of mesothelial involvement in repair processes stems from studies reporting mesothelial cell contribution to peritoneal and liver repair [17, 27]. The omentum, the peritoneal flap surrounded by mesothelial cells, has been successfully employed in various surgical repair studies, including myocardical infarction in dogs and pigs, and 5/6 nephrectomy in mice [2, 28, 29]. This is further supported by recent work from the Mutsaers lab showing that mesothelial cells isolated from the human pericardial cavity, and from rat omentum have the potential to undergo adipogenic and osteogenic differentiation [5]. These findings indicate that mesothelial cells share many properties with mesenchymal stem/stromal cells (MSCs), including the ability to promote tissue repair and a capacity for multilineage differentiation. Interestingly, a recent study has shown that the transcriptional profile of a subset of MSCs most closely resembles that of primary mesothelial cells, suggesting that some MSCs could possibly be derived from a mesothelial progenitor [30]. Here, we report that following long-term culture, mesothelial cells isolated from adult mouse omentum showed reduced expression of epithelial markers, but maintained their mesenchymal characteristics. Early passage mesothelial cells displayed clonogenicity, expressed stem cell markers and similarly to mesenchymal stem/stromal cells (MSCs), had the capacity to undergo differentiation towards the osteoblast and adipocyte lineages. However, in contrast to MSCs, which disrupt kidney development when incorporated into mouse kidney rudiment chimaeras *ex vivo* [31], we demonstrate that mesothelial cells do not inhibit nephrogenesis.

## Material and Methods

### Isolation of omentum-derived peritoneal mesothelial cells

Mice were held under an institutional licence (PEL 40/2408), approved by the local Animal Welfare Committee, at the University of Liverpool, following Home Office (UK) regulations. Mice were euthanised with carbon dioxide following Home Office (UK) regulations. Pregnant mice were ordered in from Charles River (UK), therefore no other regulated procedures were performed on mice for this project. The stomach-spleen complex was dissected out from CD1 female mice into pre-warmed mesothelial cell medium (MCM) containing DMEM (D5796, Sigma-Aldrich) supplemented with 10% FBS (F6178, Sigma-Aldrich), 100 μg/ml streptomycin, 100 U/ml penicillin (P4333, Sigma-Aldrich). The omentum explants were isolated and cultured as previously described [32]. In short, omentum tissue was isolated and any fat, blood vessels and attached cells were removed. Omentum explants were generated by cutting the compacted omentum into tightly packed pieces with diameters of between 300 and 800 μm, and seeding these into MC medium in 3.5 mm (Nunc) dishes. Attached explants were allowed to expand in conditioned media. After 14 days (d) explants and surrounding mesothelial cells (MCs) were trypsinised (10x trypsin, T4174, Sigma-Aldrich) into small dishes containing conditioned media; this was defined as passage 1 (P1). Once near-confluent MCs were trypsinised and transferred into large dishes with standard MC media. Twelve independent mouse mesothelial cell cultures were isolated with highly similar morphology (not shown); data presented here have been generated with 3 of the 12 cultures we isolated. MCs and mesenchymal stem cells (MSCs; D1 ORL UVA [D1] (ATCC® CRL-12424™)) were sub-cultured every 2–3 d in MCM at 37°C in 5% CO_2_.

### Generation of conditioned medium

Passaged MCs growing at a density of 70–80% were cultured in fresh medium for 24 hours (h). Subsequently, the supernatant was centrifuged at 1000 rpm to remove any cell debris and stored at 4°C until use. Conditioned medium was generated by adding fresh pre-warmed media at a 1:1 ratio to spin down supernatant.

### Labelling of MCs with GFP lentivirus

MCs were grown in a 24 well plate to 60% confluency. Medium was replaced with fresh medium containing polybrene (8 μg/ml). MCs were transduced with the lentivirus pLNT-SFFV-GFP with multiplicities of infection (MOI) of between 4 and 6, depending on the viral titer. Medium was replaced 24 h post-transduction and cells left to grow for a further 48–72 h. Transduced cells were cultured at 37°C in 5% CO_2_ until ready to be used for co-culture or FACS analysis.

### Flow cytometry

Fluorescence-activated cell sorting (FACS) using the 488 nm laser of a FACSAria II sorter was performed to isolate GFP-expressing MCs. Forward- and side-scatter characteristics determined the exclusion of dead cells. A yield of 88% lentivirus-labelled GFP+ MCs (MC^GFP+^) was obtained.

### Determination of population doubling time

After a homogeneous population of cobblestone mesothelial cells was achieved at passage 4 (P4), cells were seeded in triplicate at a density of 6 x 10^5^ in a 10 cm dish (Corning). At 90% confluence cells were trypsinised and counted using the trypan blue exclusion assay in a TC20™ Automated Cell Counter (BioRad). The population doubling time (PDT) was calculated using the following equations: N1 = N0*2t/T and T = t*ln(2)/(ln(N1)-ln(N0)), where N1 is the cell number of harvested cells and N0 is the cell number at the start of the incubation. T is the doubling time and t is the culture duration.

### Clonogenic assay

Mesothelial cell clones (MC clones) were generated by dilution cloning assay, whereby P5 MCs were seeded into 96-well culture plates (Nunc) at a density of 2 cells/well in conditioned medium. Wells containing one colony were identified after 24 h, and left to grow until 80–90% confluency. Cells of single colonies were subcultured into larger dishes for further passages and analysis. Images were taken using a Nikon Eclipse TS100-F. The clonogenic assay was carried out using 3 independently derived MC cultures.

### Immunofluorescence

MCs and MC clones were seeded at 4 x 10^4^ cells/chamber in an 8 chamber slides (Lab-Tek™ II, Nunc); and cultured to 80% confluence. The cells were fixed in 4% Paraformaldehyde (PFA) (P6148, Sigma-Aldrich) and permeabilised in 0.25% Triton-X (93426, Fluka), followed by blocking in 2% bovine serum albumin (BSA; BPE9701, Fisher Scientific) and incubation with primary antibodies over night at 4°C. Cells were incubated with secondary antibodies and mounted in anti-fade mounting medium Gelmount (1798510, Biomedia). Samples were documented using a Leica DMR-HC microscope with Leica DFC350FX camera and Leica application software. Experiments were performed for 3 independent biological samples.

The following primary antibodies were used: Rabbit polyclonal ZO1 (1:200, 40–2200, Life Technologies), mouse monoclonal Wt1 (1:100, 05–753, Millipore), mouse monoclonal α-smooth muscle actin (1:200, A2547, Sigma-Aldrich), rat monoclonal Pecam (1:100, 550274, BD Pharmingen), rabbit polyclonal cytokeratin (1:200, Z0622, Dako), rabbit polyclonal Bmi1 (1:50, AP8756a, Abgent), goat polyclonal Sox9 (1:100, SC-20095, Santa Cruz), goat polyclonal vimentin (1:200, 64740, ICN Biomedicals Inc.), rabbit polyclonal GFP (1:5000, ab290; Abcam), mouse monoclonal Megalin IgG1 (1:200, DM3613P, Acris), rat monoclonal Laminin α1β1 (1:200, MAB1905, Millipore), rabbit polyclonal Pax2 (1:200, PRB-276P, Biolegend), rabbit polyclonal Six2 (1:200, 11562-1-AP, Proteintech). Secondary antibodies used were: Alexa Fluor 488 coupled (AF488) goat anti-mouse (1:1000, A11001, Life Technologies), AF488 goat anti-rabbit (1:1000, A11008, Life Technologies), AF594 goat anti-mouse (1:1000, A11032, Life Technologies), AF594 goat anti-rat (1:1000, A11007, Life Technologies), AF647 goat anti-rabbit IgG (1:1000, A21245, Life Technologies) AF488 donkey anti-Rabbit IgG (1:1000, A-21206, Life Technologies), AF546 donkey anti-goat IgG (1:1000, A-11056, Life Technologies) and nuclear counterstain DAPI (1:1000, D1306, Life Technologies).

### Adipogenic and osteogenic differentiation assays

For adipogenic and osteogenic differentiation, MCs at P5, 13 and 26 and MSCs were seeded at 1000 cells/well in a 12 well plate and cultured in adipogenic medium (AM) containing MC medium supplemented with 100 nM dexamethasone (D4902, Sigma-Aldrich), 500 nM 3-isobutyl-1-methylxanthine (I7018, Sigma-Aldrich), 50 μM indomethacin (I7378, Sigma-Aldrich) and 1 μg/ml insulin (I6634, Sigma-Aldrich) or osteogenic medium (OM) containing MC medium supplemented with 100 nM dexamethasone, 10 mM β-glycerophosphate (G9422, Sigma-Aldrich) and 25 μg/ml of 2-Phospho-L-ascorbic (49752, Sigma-Aldrich) or untreated standard MC medium for 14 d. To visualise differentiation, cells treated in AM were fixed in 4% PFA for 10 minutes (min), washed twice in 60% isopropanol for 5 min, and stained in 0.5% oil red in isopropanol for 10 min at room temperature. Cells treated in OM were stained in 2% alizarin red (pH4.5) for 2 min. Samples were imaged on a Nikon Eclipse TS100-F. Each assay was run with 3 independent biological samples.

### RT-PCR and qPCR

Total RNA was extracted from a 10 cm dish of confluent cells using 1ml Trizol reagent (15596018, Life Technologies), and 1.4 μg of total RNA was reverse transcribed to cDNA using superscript III reverse transcriptase Kit (18080044, Life Technologies). Gene transcription was detected by real-time PCR with the CFX Connect™ Real-Time PCR Detection System (BioRad), using specific primers designed in-house and Blast searched (S1 Table). Amplified cDNAs were either run on a 1% agarose gel to document gene expression or analyzed by the CFX manager software (BioRad) to compute fold change in expression relative to a control. Reactions were run with the following cycling conditions: 50 cycle of 95°C for 3 min initial polymerase activation followed by 50 cycles of, 95°C for 10 seconds (sec) and 60°C for 30 sec. qPCR specificity was assessed using melt curves and agarose gels to study PCR product band sizes. Target values were normalized against two housekeeping genes GAPDH and β-Actin using the relative quantification method with n = 3 independent biological samples per condition.

### Kidney re-aggregation chimera assay

The embryonic kidney re-aggregation assay was based on the Unbekandt and Davies (2010) protocol [33]. Briefly, embryonic day (E)13.5 mouse kidneys were harvested and dissociated into single cells following 10 min incubation in 0.25% trypsin/PBS (T4174, Sigma-Aldrich) with intermittent gentle agitation. We had explored the use of a cell strainer in preliminary experiments but found that it resulted in loss of viable cells. After centrifugation at 3000 rpm for 2 min, cells were re-suspended in MEME (M5650, Sigma-Aldrich) containing 10% FCS, and single embryonic kidney cells were counted using a haemocytometer.

An average of 60 embryonic kidneys (depending on litter size) were collected for each experiment, dissociated and counted before re-aggregates were set up using 200K cells per sample or 180K embryonic kidney cells mixed with 20K MC^GFP+^ (P22-32) at 1:10 ratio. On average, 16 pellets were generated and cultured for each experiment. Re-aggregated chimeric rudiments (MC rudiments), re-aggregated control rudiments (rControl rudiments) or whole embryonic kidney rudiments (eControl rudiments) were cultured on Millipore filters (RTTP02500) placed on metal grids at the liquid-air-interface. The rudiments were fixed in 4% PFA for 30 min at 7 d post seeding (embryonic age E13.5 +7 d). For immunofluorescence analysis, samples were blocked in 10% serum, 1% Triton-X in PBS, followed by incubation in primary and secondary antibodies, and subsequent mounting in 80% glycerol for viewing on the Leica TCS MP2 AOBS confocal microscope.

### Isolation and analysis of GFP+ MCs from the kidney rudiment assay

Between 8–17 chimeric rudiment pellets were harvested following for 7 d of culture and trypsinised in 2x diluted (PBS) 10x trypsin (T4174, Sigma-Aldrich) for 10 min with intermittent and gentle agitation. Once the rudiments were fully disaggregated, the single cell suspension was counted using a haemocytometer and their viability checked. Next, the cells were pelleted at 1000 rpm for 5 min and re-suspended in PBS containing 10% FBS. Using the BD FACS ARIA III, MC^GFP+^ cells were harvested from the single cell mix through a number of gating parameters that were initially set up using non-aggregated MC^GFP+^. Specifically, healthy cells were identified by plotting side scatter (SSC-A) against forward scatter (FSC-A). Next, cell clusters were gated out using FSC-A versus (vs) FSC-H and SSC-W vs SSC-H plots. GFP-positive cells were selected by plotting the excitation signal from the cells with a 488-nm laser (GFP FITC-A log) vs SSC-A. Finally, count vs GFP FITC-A parameters were used to plot a histogram from which the highest fluorescence cells were sorted.

### Data analysis

Immunofluorescence and bright field images were prepared on Photoshop and Illustrator CS6. The Bio-Rad CFX manager was used to perform qPCR data analysis, and quantitative data was analysed using Excel 2013. Statistical significance was determined using either a one way ANOVA for the analysis of variance and was followed by Sidak’s post-hoc multiple comparisons test for variance among the groups; or a Student’s t-test. Statistical analysis was performed using GraphPad Prism version 6.00 for Windows. A p-value <0.05 was considered significant.

## Results

### Long-term culture of omentum explant-derived mesothelial cells

**Fig 1:**
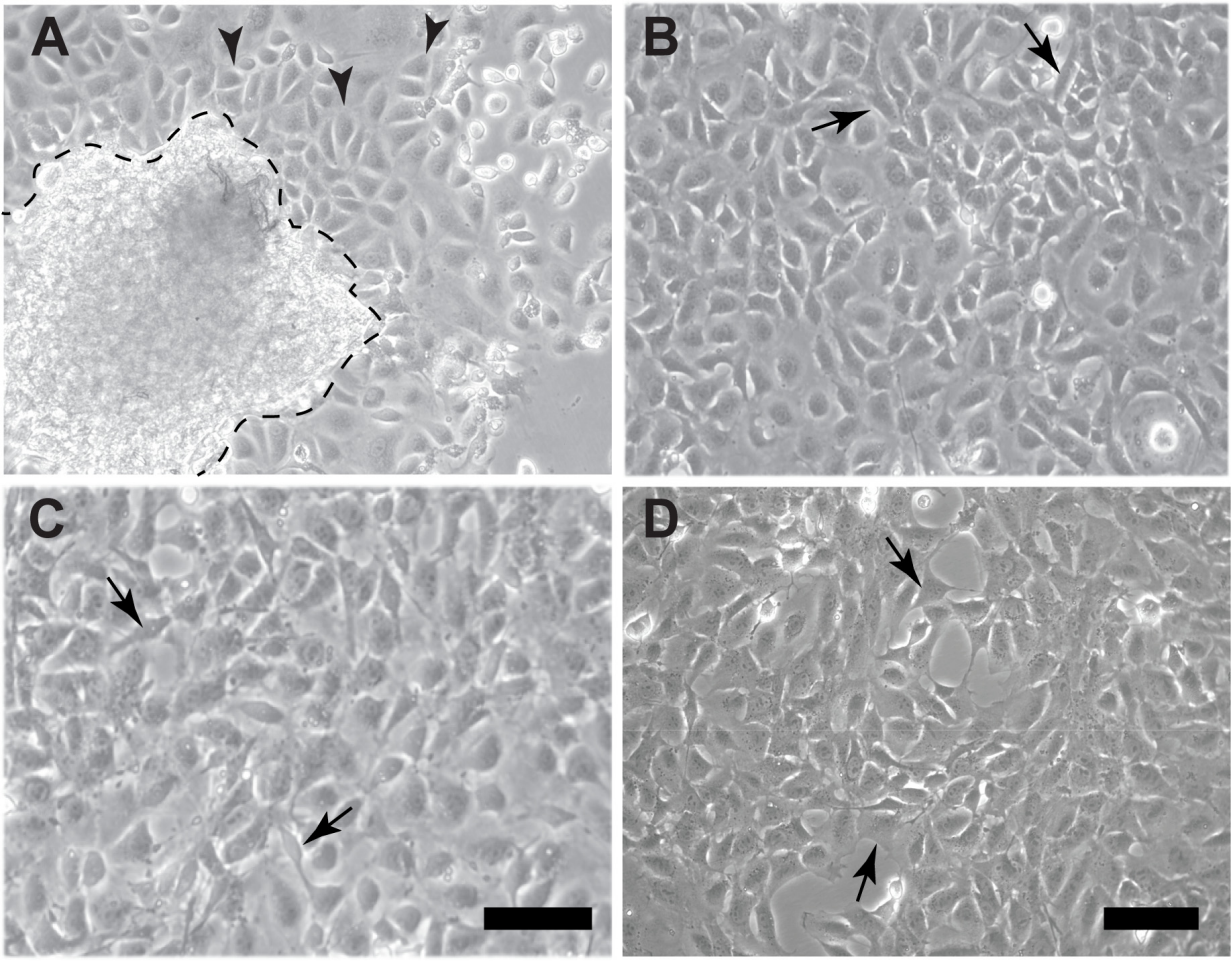
Generation of omentum-derived adult mouse mesothelial cell lines. (A) Following 7 days of culture, primary MCs had migrated out of an omentum explant (outlined by stippled line). MCs in these cultures had a typical cobblestone-type appearance (arrowheads). (B-D) Passaged MCs overall retained their epithelial phenotype even though some cells adopted a slightly elongated shape (arrows) (B, P1). This appearance was un-affected by passage number (C, P5; D, P24). Scale bar represents 50 μm (A, B, D; C).

Previously we had described the culture of omentum explants from adult mice for up to 5 days where mesothelial cells (MCs) grew out from the explant to form an epithelial sheet at around d2 post seeding [32]. At d5 post seeding, cells at the edge of the outgrowing sheet appeared to have a more mesenchymal phenotype, while cells closer to the original explant remained epithelial. We have now generated twelve mesothelial cell cultures by trypsinising at around d14 the cultured omentum explants including any MCs that had moved away from the explant (Fig 1A). The resulting passage 1 (P1) MCs displayed a typical cobblestone phenotype, indicating epithelial characteristics (Fig 1B); however, some cells adopted a slightly elongated shape. This morphology remained largely unchanged even at higher passages (Fig 1C and 1D).

Population doubling times (PDT) for MCs between P5 to P16 were between 20 and 40 h, stabilising at around 24 h between P8 and P16 (S1 Fig). However, we found that at around P36, the PDT of MCs had slowed to around 40 h (data not shown).

**Fig 2:**
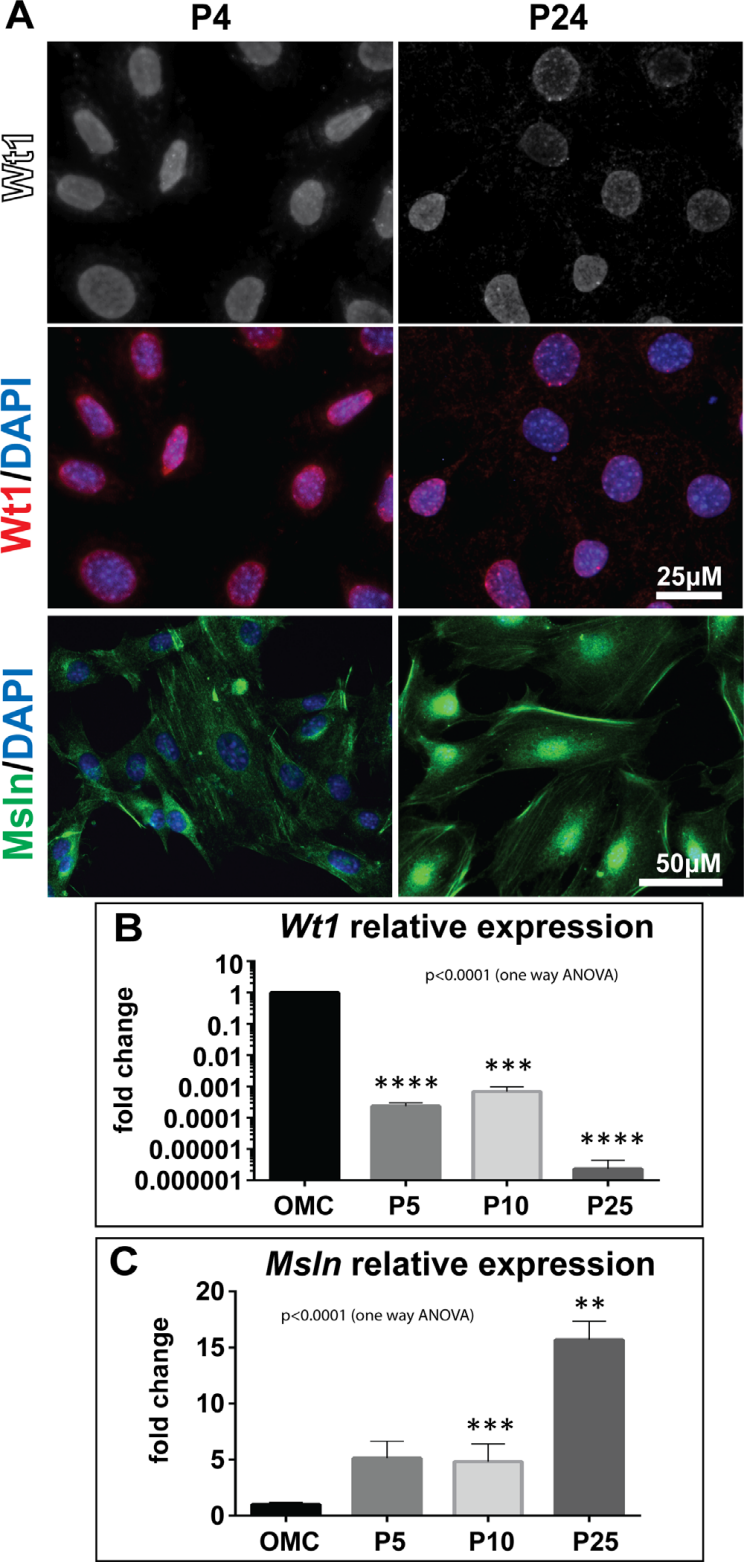
Expression of mesothelial markers over passages. (A) Immunofluorescence analysis of Wt1 and mesothelin (Msln) in P4 to P24 MCs showed that expression of both proteins was present throughout passages. Scale bars represent 25 μm (Wt1/DAPI) and 50 μm (Msln/DAPI). (B) qPCR analysis of mRNA expression for *Wt1* showed significant downregulation in cultured P5, P10 and P25 MCs relative to OMC. (C) mRNA expression of *Msln* (C) was upregulated in the cultured cells compared to OMC. Significant differences compared to OMC were determined using a one way ANOVA with Sidak’s multiple comparison test where; ****p < 0.0001, ***p < 0.001 and **p < 0.01.

In order to assess whether MCs maintained their mesothelial characteristics throughout passages, we performed immunofluorescence (IF) and gene expression analysis for the two mesothelial markers, Wilms tumour protein 1 (Wt1) and mesothelin (Msln). Wt1 was detected in the nuclei of P4 and P24 MCs (Fig 2A). Mesothelin expression was found in P4 MCs predominantly in the cytoplasm, while in P24 cells the protein was also detected in the nucleus and the cell membranes (Fig 2A). Interestingly, qPCR analysis showed that relative expression of *Wt1* decreased significantly with increasing passages when compared to cultured omentum explants (OMCs) (Fig 2B; S2 Table), while Msln expression levels were significantly upregulated with increasing passages (Fig 2C; S2 Table).

**Fig 3:**
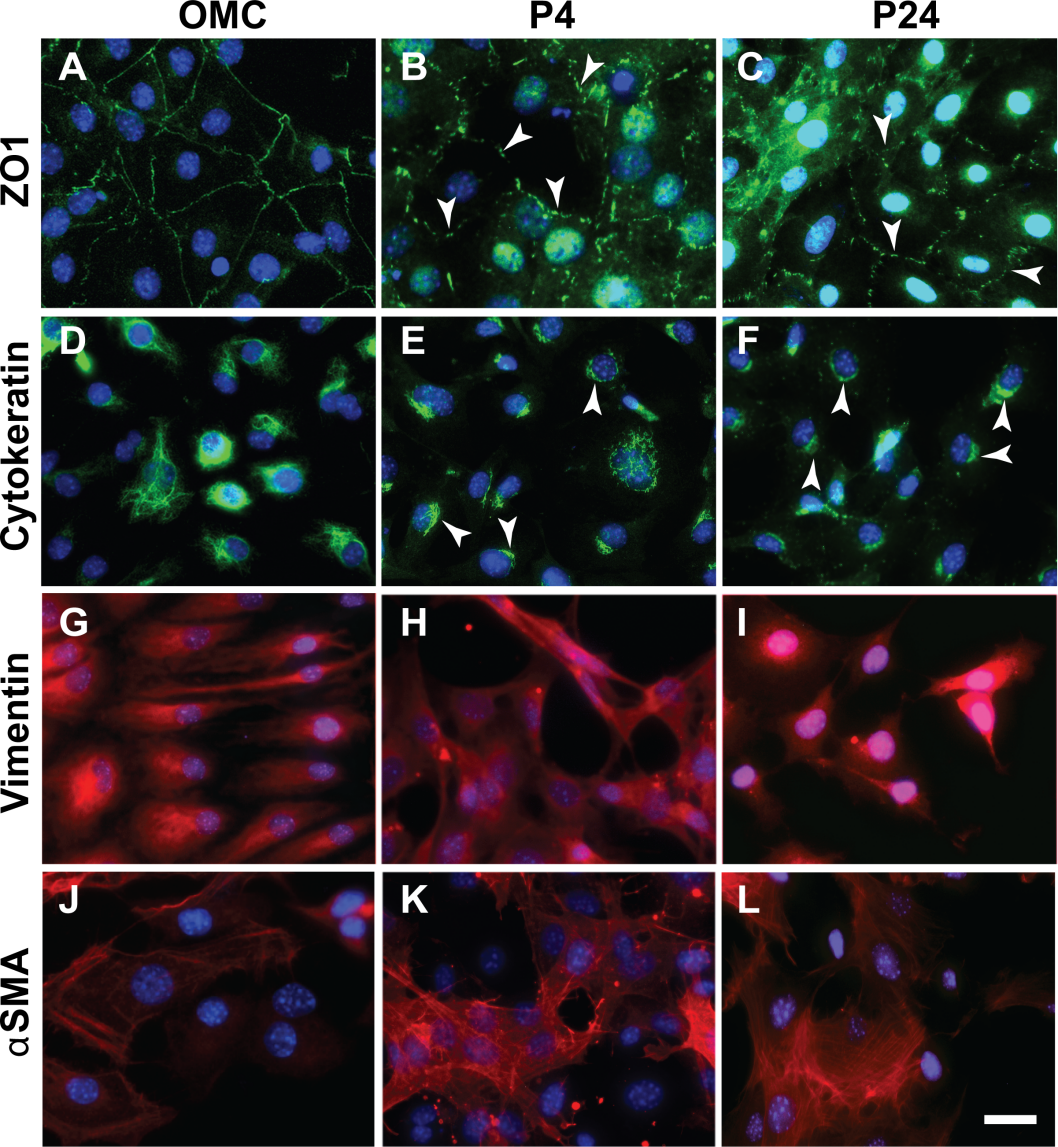
Cultured mesothelial cells expressed both epithelial and mesenchymal characteristics by immunofluorescence. (A) OMC cells showed strong ZO1 tight junctional bands at the cell-cell contacts, while ZO1 expression had a punctate appearance (arrowheads) at the cell perimeter In P4 and P24 MCs (B-C). (D) Cytokeratin intermediate filaments localised across the cytoplasm in OMC cells, while in P4 and P24 MCs the expression was reduced to the perinuclear regions (arrowheads; E-F). Expression of Vim (G-I) and αSMA (J-L) was similar between OMC, P4 and 24 MCs. Scale bar 50μM (A-L).

To further characterise MCs at low and at high passage, we performed IF and qPCR analysis for a range of epithelial and mesenchymal markers. While the tight junctional marker ZO1 was localised continuously around the cell perimeter in MCs grown out of omentum explants (Fig 3A), it appeared in a punctate pattern around the perimeter of P4 and P24 MCs (Fig 3B and 3C). Cytokeratin intermediate filament protein networks were seen across the cytoplasm and in the perinuclear region of OMC cells (Fig 3D). However, in the P4 and P24 MCs, cytokeratins were localised mostly in the perinuclear region (Fig 3E and 3F). We detected the mesenchymal protein Vimentin (Vim) throughout the cytoplasm in OMC, P4 and P24 MCs (Fig 3G–3I), while alpha smooth muscle actin (αSMA) was expressed at varying levels in MCs of all three passages (Fig 3J–3L). The endothelial marker PECAM was not detectable in any of our samples (not shown).

**Fig 4:**
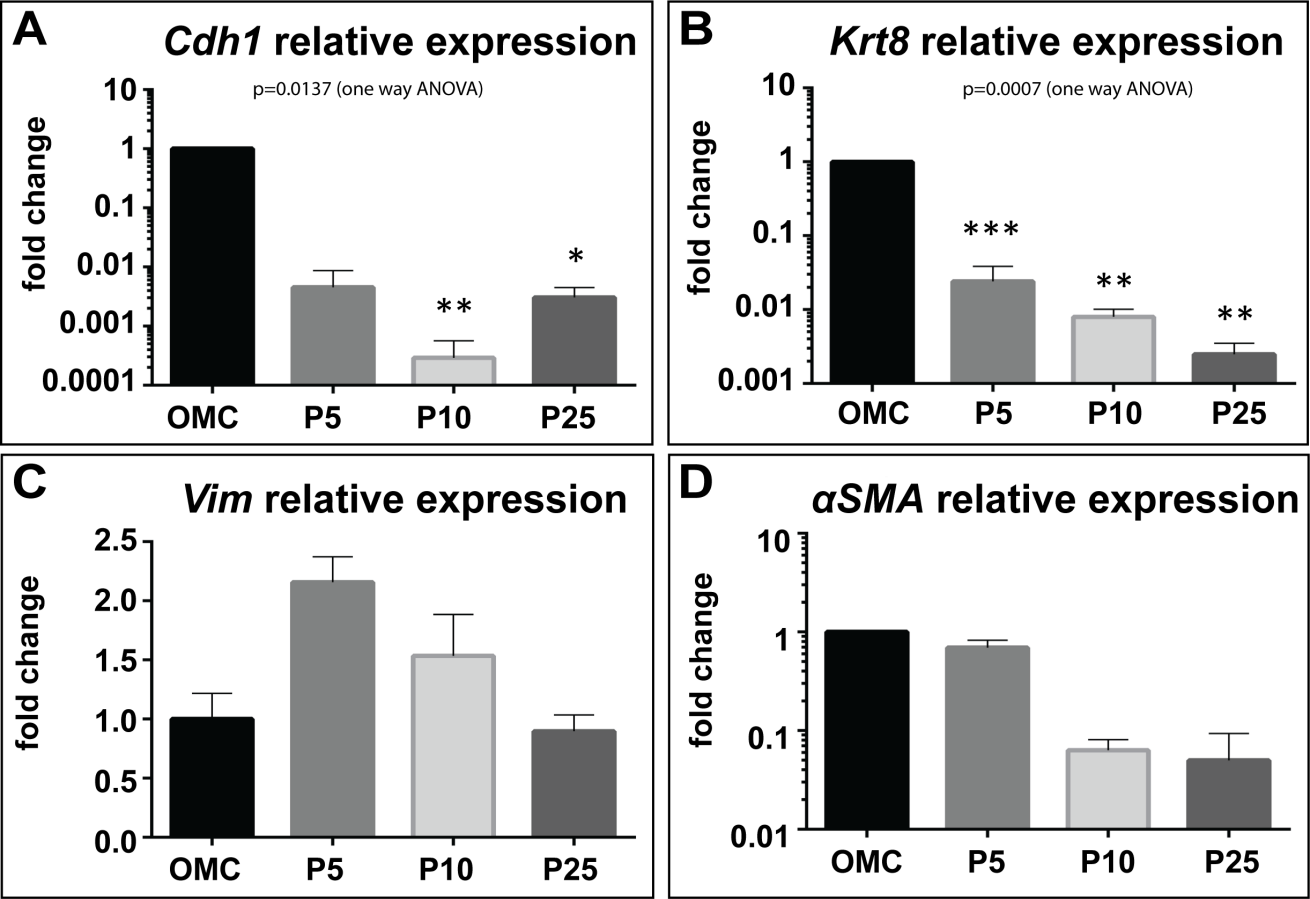
Long-term culture influenced epithelial gene expression. Analysis of mRNA expression levels for the epithelial genes *Cdh1* (A) and *Krt8* (B) showed significant down regulation in P5, P10 and P25 MCs relative to OMC. By contrast, expression of mesenchymal markers *Vim* (C) and *αSMA* (D) showed no significant change in mesothelial cell cultures of increasing passage. Significant differences compared to OMC were determined using a one way ANOVA with Sidak’s multiple comparison test where; ***p < 0.001, **p < 0.01, and *p<0.05.

Expression analysis by qPCR of the epithelial markers E-cadherin (*Cdh1*) and cytokeratin 8 (*Krt8*) showed that in the passaged MCs, mRNA levels of both epithelial genes significantly decreased with increasing passages when compared to omentum cultures (Fig 4A and 4B, S2 Table). Interestingly, relative expression of both *Vim* and *αSMA* was not significantly different between omentum culture and passaged MCs (Fig 4C and 4D; S2 Table).

These results suggested that the molecular signature of the MCs was changed in response to repeated passaging, possibly indicating dedifferentiation processes of the cells in culture.

### Mesothelial cells were clonogenic

**Fig 5:**
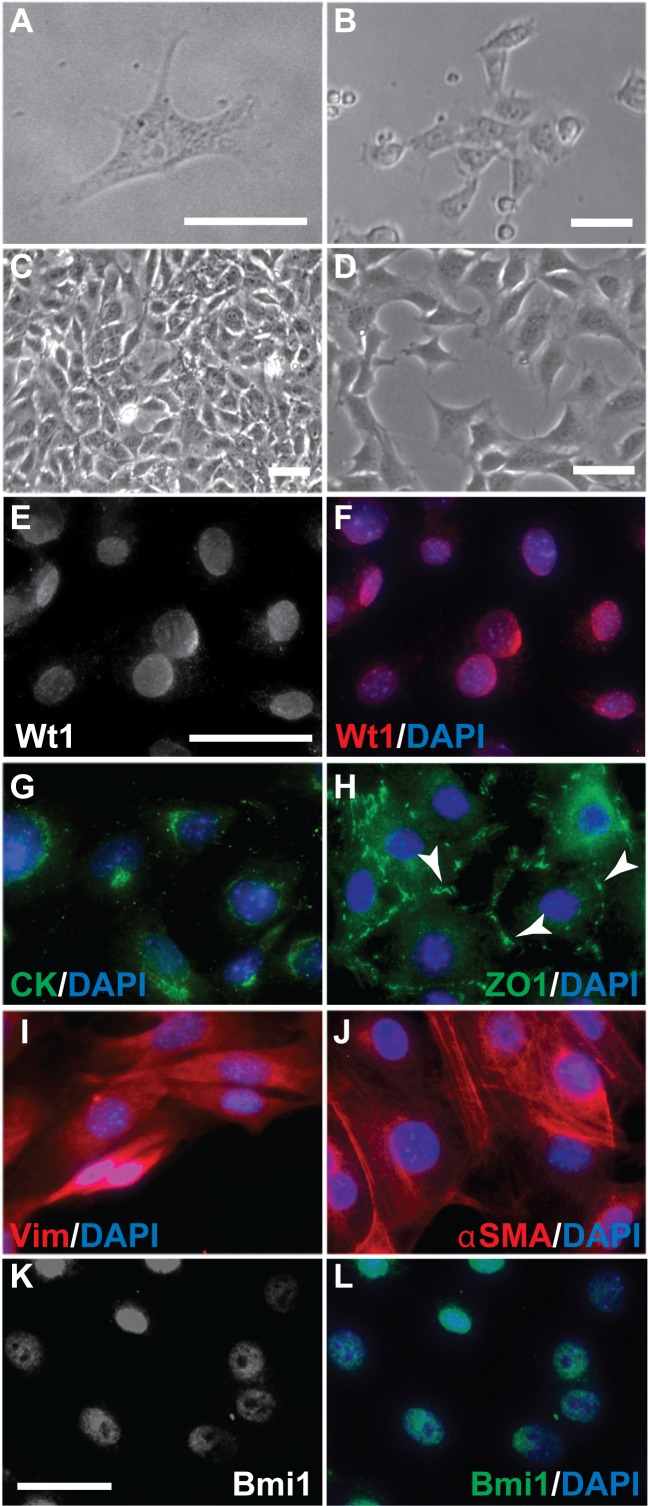
Clonogenic potential of mesothelial cells. (A) Single cell from which one of the clones was started by dilution cloning. (B) MC clone at 2 weeks after cloning. (C) MC clone at confluence. (D) MC clone cells after passaging. Immunofluorescence staining for the mesothelial marker Wt1 (E-F), epithelial markers Cytokeratin (CK) (G) and ZO1 (H), and the mesenchymal markers Vimentin (Vim) (I) and α smooth muscle actin (αSMA) (J) in cells of mesothelial cell-derived clones revealed expression patterns similar to uncloned cells. The stem cell marker Bmi1 was detected in clonal MCs (K-L). Scale bars are 50 μm (A, B, C, D, E-J, K-L).

Dedifferentiation of cells can be an indicator of the acquisition of stem or progenitor cell status [34]. To determine whether cultured MCs isolated from adult omentum had stem or progenitor cell properties, we analysed their clonogenic potential. Using dilution cloning, we generated single cell clones from P5 MCs. On average, up to 4 clones formed per independent omentum-derived MC culture (Fig 5A–5D). Cells from all clones had the typical morphological appearance of passaged MCs and have been successfully cultured for more than 20 passages. MCs cultured from clones expressed Cytokeratin, ZO1, Vim, αSMA, and Wt1 in a distribution similar to P4 and P24 MCs (Fig 5E–5J).

### Cultured mesothelial cells expressed stem cell markers

**Fig 6:**
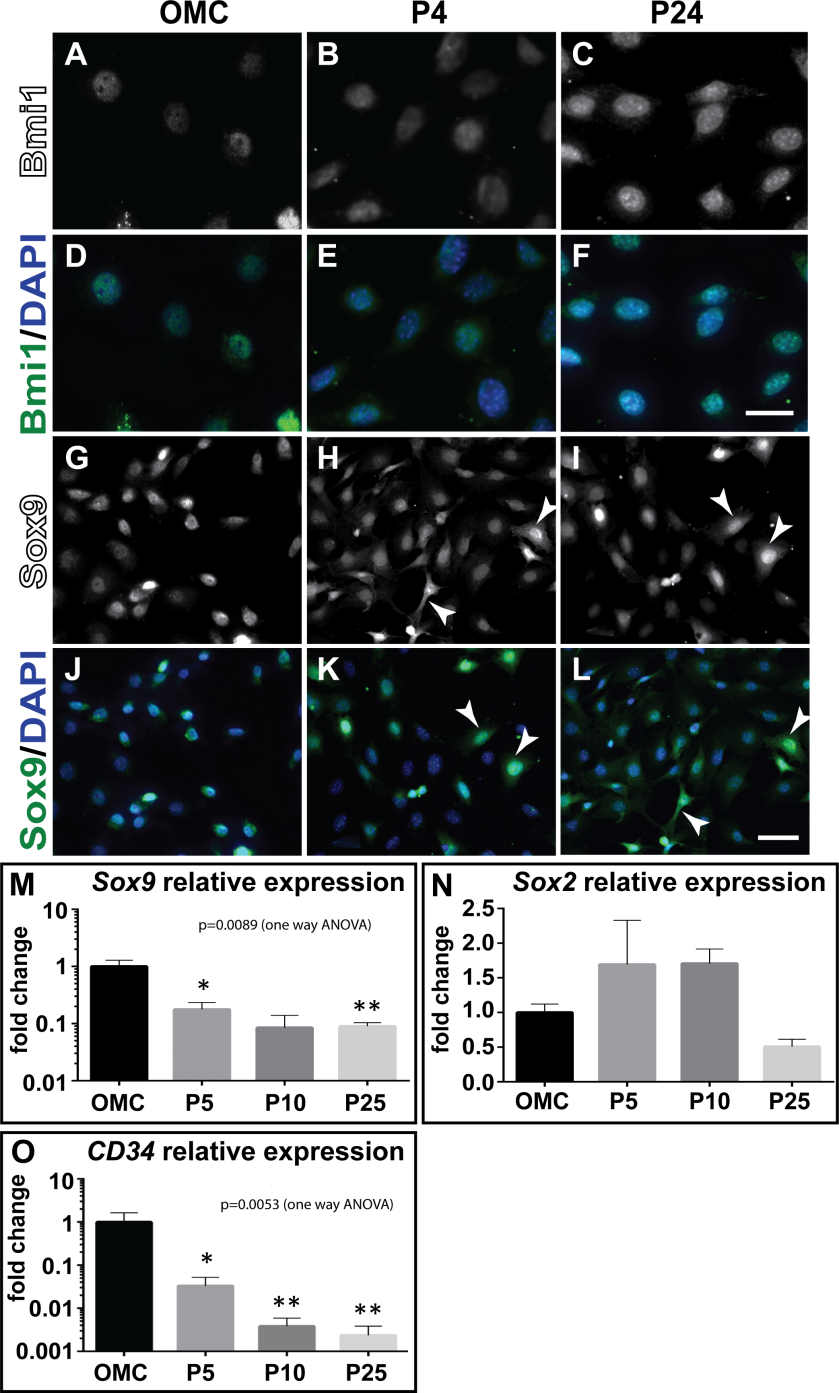
Mesothelial cells expressed stem/progenitor cell markers. Bmi1 and Sox9 nuclear localisation was detected through immunofluorescence in OMC (A, D; G, J), P4 (B, E; H, K) and P24 cells (C, F; I, L), respectively. Relative expression of stem cell markers in passaged MCs was maintained for *Sox2* (N), while *Sox9* (M) and *CD34* (O) were downregulated in the passaged cells, respectively. Significant differences compared to OMC were determined using a one way ANOVA with Sidak’s multiple comparison test where; **p < 0.01, and *p<0.05. Scale bars are 25 μm (A-F) and 50 μm (G-L).

Next, we determined whether the cultured MCs expressed stem cell markers. Using IF, we could identify nuclear expression of the stem cell markers Bmi1 and Sox9 in primary MCs of omentum cultures and in P4 and P24 MCs (Fig 6A–6L). Bmi1 nuclear expression was also detectable in the clonal MCs (Figs 5K and 6L). More specifically, nuclear Bmi1 expression was strongest in the P24 MCs (Fig 6C and 6F). By contrast, Sox9, in addition to nuclear localisation, showed cytoplasmic expression in cultured MCs (Fig 6H, 6K and 6I, 6L).

Using qPCR, we analysed the relative expression of the stem cell markers *Sox9*, *Sox2* and *CD34* (Fig 6M–6O). While *Sox2* expression was maintained at similar levels between omentum cultures and passaged MCs, *Sox9* and *CD34* were downregulated with increasing passages.

### Mesothelial cells showed adipogenic and osteogenic differentiation potential

**Fig 7:**
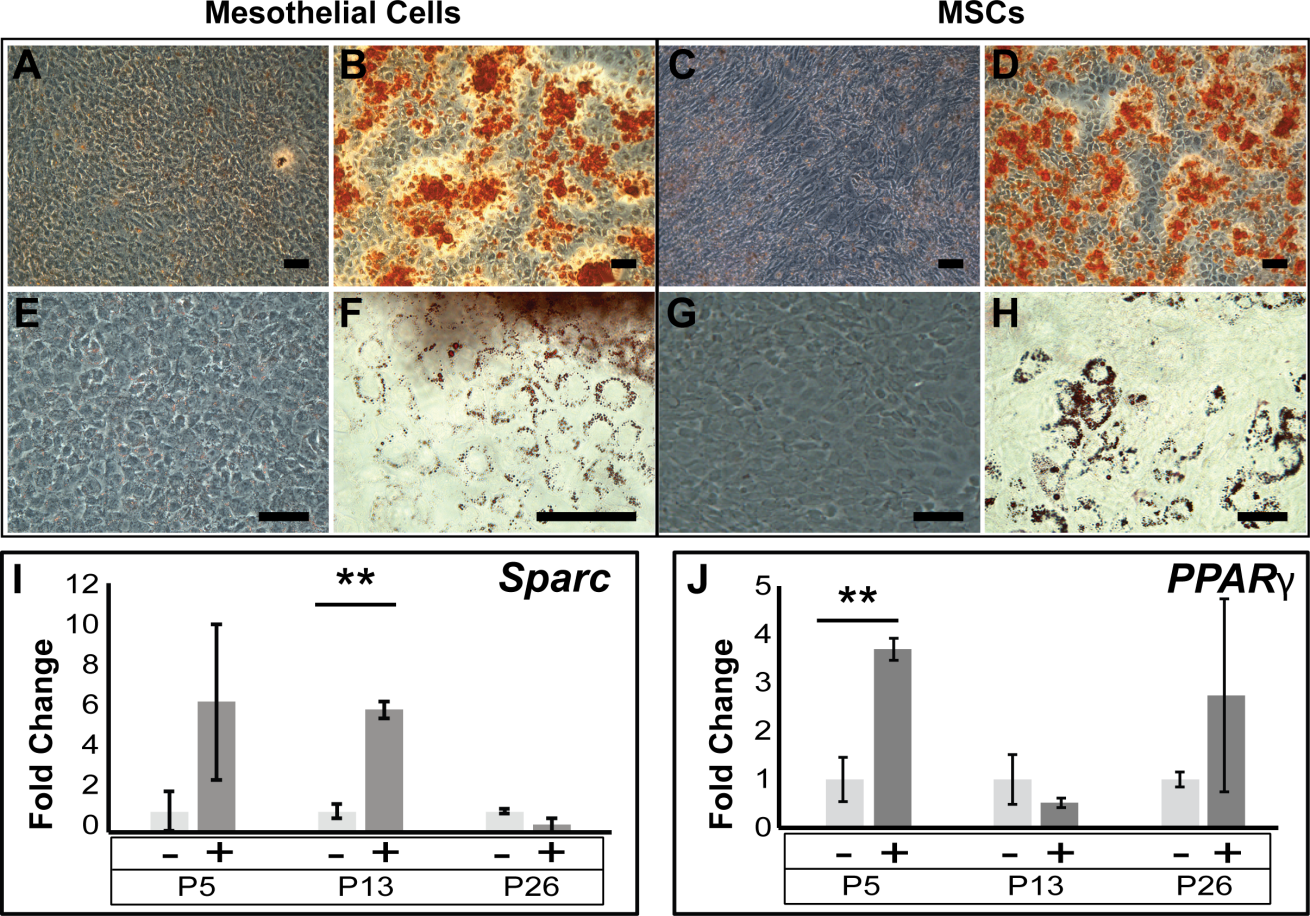
Analysis of osteogenic and adipogenic potential. Using Alizarin S red staining, Calcium deposits could be detected in P13 MCs (B) and P21 MSCs (D), indicating osteogenic differentiation, while cells under control conditions failed to exhibit the deposits (A, C). Fat droplet accumulation could be detected in P5 MCs (F) and, slightly more pronounced in MSCs (H). Control conditions showed no generation of fat droplets (E, G). Expression analysis by qPCR revealed that the osteogenic marker *Sparc* was up-regulated in the earlier passages (I), with a significant 6-fold change in P13 MCs. A significant 3.7-fold increase in expression was observed in P5 MCs for the adipogenic gene *PPARγ* (K). A Student’s t-test was used to calculate significance. Scale bars are 30 μM.

To assess whether the cultured mesothelial cells possessed multi-lineage differentiation potential, we subjected the cells to osteogenic and adipogenic differentiation conditions. MCs generated a robust response to the osteogenic culture conditions, producing calcium deposits that could be seen through phase contrast microscopy (data not shown) and stained positive with Alizarin Red S (Fig 7B), similarly to the osteogenic changes observed in mouse mesenchymal stem cells (MSCs) which we used as positive control (Fig 7D). After culture in adipogenic conditions, MCs at P5 but less so at P13 and P26, produced oil red-stained fat vacuoles (Fig 7F and not shown); however these were more dispersed when compared with MSCs (Fig 7H). We analysed the adipogenic and osteogenic differentiation of early, medium and high passage MCs by qPCR for two specific markers. Our results showed that in response to osteogenic culture conditions, MCs at medium passage (P13) significantly upregulated the mRNA expression of *secreted acidic cysteine rich glycoprotein* (*Sparc*, *Osteonectin*) (Fig 7I), which is important for osteoblast maintenance [35]. Furthermore, we found that the mRNA expression of the master control protein for adipogenesis, *peroxisome proliferator-activated receptor gamm*a (*PPARγ*) [36, 37] was significantly increased in early passage MCs under adipogenic conditions (Fig 7J). These results suggest that MCs cultured up to passage 13 mostly maintained the potential to respond to osteogenic stimulation by change of phenotype and specific marker expression, while the adipogenic response was only maintained during early passages.

### Mesothelial cells do not inhibit nephrogenesis *ex vivo*

We have previously shown that surprisingly, MSCs disrupt the development of mouse kidney rudiments *ex vivo* [31]. Given that MCs share some properties with MSCs, we investigated whether MCs also had a negative impact on kidney development, or whether MCs, unlike MSCs, could integrate into developing nephron structures, by using a modified chimeric embryonic kidney rudiment assay [33]. Re-aggregated E13.5 embryonic kidney rudiments (rControl rudiments) gave rise to developing nephrons including proximal tubules, similar to whole embryonic kidney rudiments (eControl rudiments) (S2 and S3 Figs).

**Fig 8:**
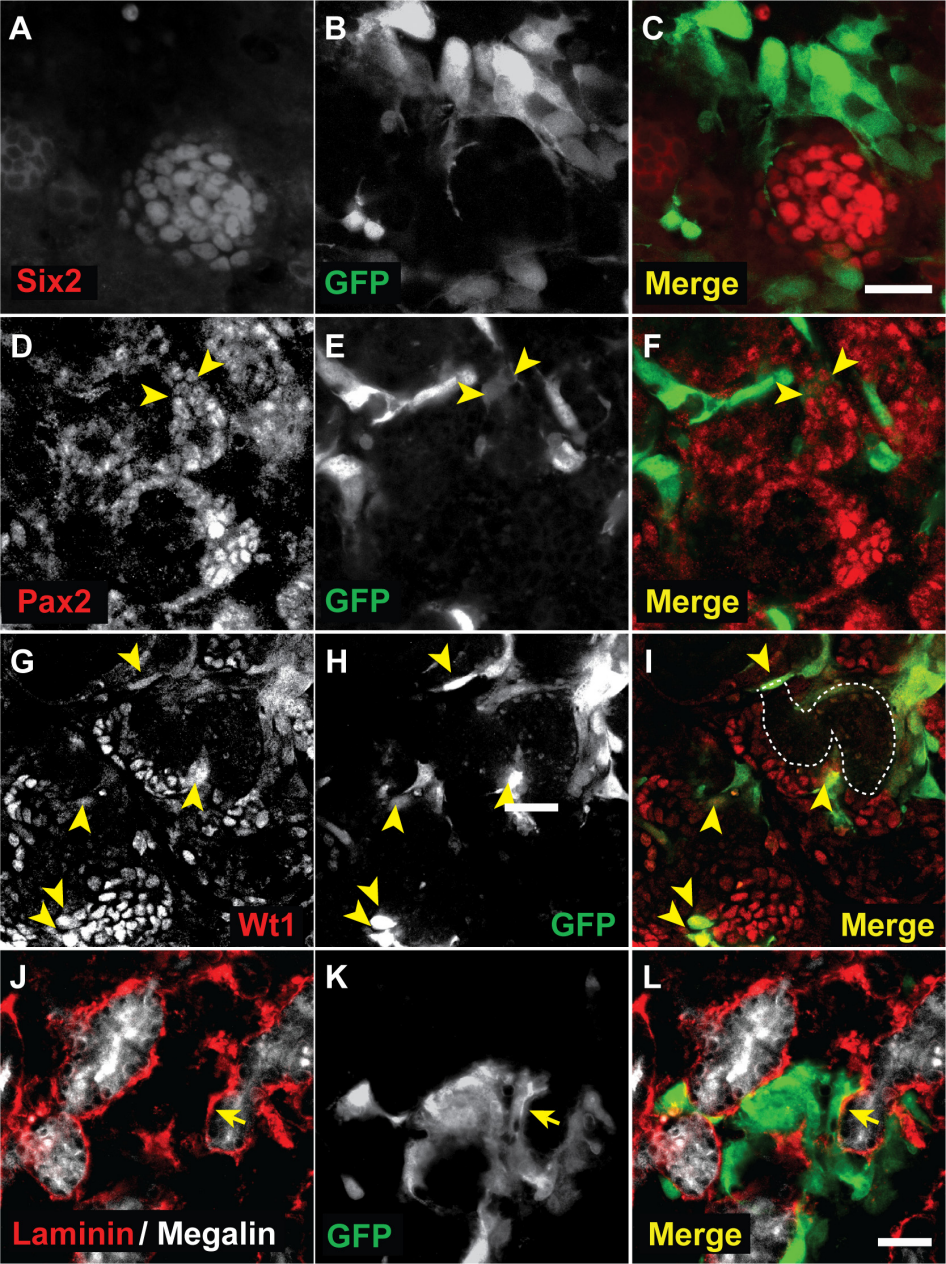
Mesothelial cells are found within the developing kidney rudiment. MC^GFP+^ were seen situated around Six2-expressing cells in the cap mesenchyme (A-C), and close to Pax2- (D-F) and Wt1- (G-I) expressing nascent nephron structures. In very few cases, Wt1- and Pax2-expression seemed to colocalise with the GFP fluorescence of the MC^GFP+^ cells (arrowheads, D-I). An S-shaped body is outlined in (I). GFP-positive MCs were found closely attached to Laminin of the basement membrane of Megalin-expressing proximal tubules (arrows, J-L). Scale bars are 50 μm (A-C, D-L).

Next, we investigated the effect of MCs on nephrogenesis *ex vivo* by generating chimeric kidney rudiments during 7 days of culture. In order to be able to identify the MCs, they were transduced with a GFP lentivirus (MC^GFP+^); expression levels of mesothelial, epithelial and mesenchymal markers including *Wt1*, *Msln*, *Cdh1*, *Krt8*, *Vim* and *αSMA*, were maintained in MC^GFP+^ cells when compared to passage-matched non-transduced cells (not shown). After mixing with embryonic kidney cells and during subsequent 7 days of culture, analysis revealed that MC^GFP+^ cells had clearly survived within the developing kidney chimera (S4 Fig). To assess whether nephron structures had formed within the chimeric rudiments, and whether MCs had contributed to the developing nephrons, we performed immunolabelling with a range of markers. Immunodetection of Six2 revealed the presence of cap mesenchyme; however, MC^GFP+^ cells were not found within the cap mesenchyme, but were instead located around the Six2^+^ cell condensates (Fig 8A–8C). Expression of Pax2, a marker for condensing mesenchyme and early nephron structures, and of Wt1, which is also expressed in the cap mesenchyme but then localises to the renal vesicle and developing glomeruli, could be detected in the chimeric rudiments, providing evidence of nephron formation (Fig 8D and 8G). Similarly to the observation with Six2, MC^GFP+^ cells were localised close to and around developing nephron structures (Fig 8D–8F and 8G–8I). In a few instances we observed GFP expression in cells within Pax2^+^ or Wt1^+^ nephron structures, however MC^GFP+^ cells were not detected in comma- or S-shaped bodies (Fig 8G–8I). Further support for nephron development in chimeric rudiments was provided by labelling for Laminin, a marker for basement membranes, and Megalin, a multi-ligand receptor specifically expressed in the proximal tubules of the kidney (Fig 8J). Interestingly, we observed that the MC^GFP+^ cells were arranged around the proximal tubule structures, directly abutting the tubular basement membrane (Fig 8K and 8L).

These results demonstrated that MC^GFP+^ cells, in contrast to MSCs, did not display any noticeable inhibitory effects on kidney development. However, while a few MC^GFP+^ cells appeared to integrate into developing nephrons, they mostly aligned with the basement membranes of the epithelial or tubular elements.

**Fig 9:**
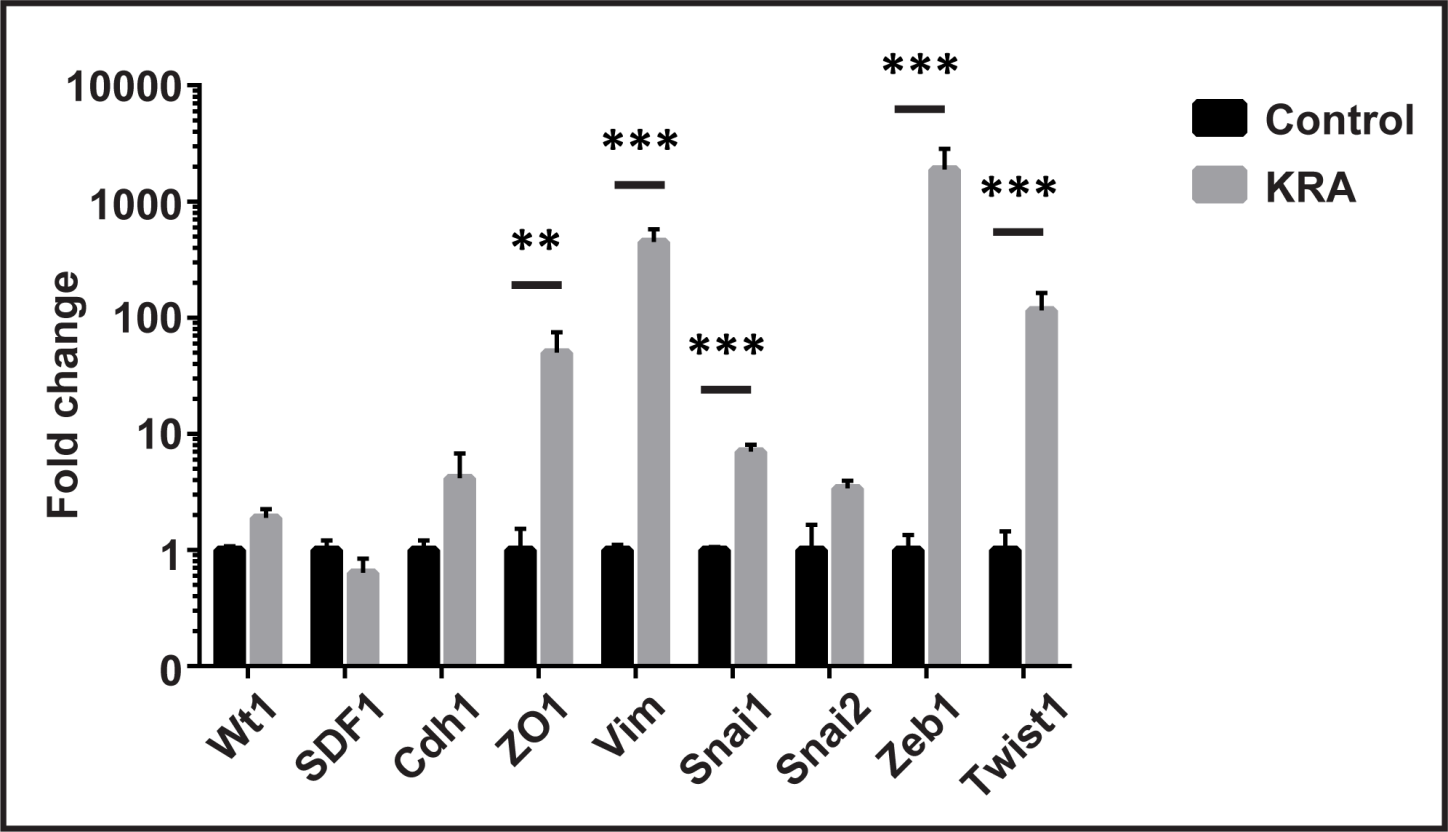
Mesothelial cells responded to the nephrogenic environment. After isolation from the chimeric rudiments (KRA), MC^GFP+^ cells were analysed by qPCR in comparison to non-treated MCs of the same passage (Control). Relative expression analysis revealed that the epithelial marker *ZO1*, the mesenchymal marker *Vim* and the EMT regulators *Snai1*, *Zeb1* and *Twist1* were significantly up-regulated, while *Wt1*, *SDF1* and *Snai2* were not significantly changed. A Student’s t-test was performed to determine statistical significance.

In order to further analyse the response of MC^GFP+^ cells to the nephrogenic environment in the chimeric kidney rudiments, we isolated the cells from rudiments by FACS after 7 days of culture and performed qPCR analysis. Interestingly, expression levels of markers involved in nephrogenesis were either not significantly changed or could not be amplified in the MC^GFP+^ cells (data not shown). Nevertheless, our analysis revealed that the nephrogenic environment stimulated the MC^GFP+^ cells to significantly up-regulate expression of *ZO1*, *Vim*, *Snail1* (*Snai1*), *Zeb1* and *Twist1* (Fig 9).

## Discussion

In this study we have analysed the stemness and differentiation potential of omentum-derived mouse mesothelial cells. We demonstrate the successful long-term culture of primary mesothelial cells. However, in response to multiple passaging, the cells shifted their expression profile, becoming less epithelial. At early passages, cultured mesothelial cells displayed stem or progenitor cell characteristics, as evidenced by the fact that they exhibited clonogenic potential, expressed stem cell markers, and showed differentiation along the osteogenic and adipogenic lineage. Furthermore, by making use of a chimeric embryonic kidney rudiment assay, we demonstrate that unlike MSCs, mesothelial cells do not have any noticeable adverse effects on the *ex vivo* development of mouse kidney rudiments.

We have previously shown that culture of tissue explants isolated from mouse adult omentum led to the outgrowth of mesothelial cells with typical mesothelial characteristics [32]. Here, we report the long-term culture of several individually isolated mesothelial cell cultures from mouse adult omentum for over 30 passages without evidence of senescence. Population doubling times stabilised to around 24 hours between passage 8 and 16. This is in contrast to human omentum-derived mesothelial cells which have been reported to undergo premature senescence [38, 39]. Characterization of the MC cultures at different passages revealed that while the overall morphology of the cells remained similar, the molecular signature of the cells changed (S5 Fig). In particular, mRNA levels of the key mesothelial marker *Wt1* were downregulated, but nevertheless, Wt1 protein could be clearly identified by immunofluorescence in the nuclei of low and high passage cells. By contrast, Msln was detectable in cells at both low and high passages using immunofluorescence, with mRNA levels increasing significantly with higher passages. Thus, the two mesothelial markers used in our analysis showed opposing responses to passaging. Loss of Wt1 in the epicardium has recently been shown not to affect expression of Msln [40], suggesting that the significant increase in *Msln* expression we have observed is independent of *Wt1* expression levels in cultured MCs.

Mesothelial cells express both epithelial and mesenchymal markers *in vivo* and have the ability to transdifferentiate into myofibroblasts in response to stress or injury, through a process called mesothelial-to-mesenchymal transition (MMT) [3, 9, 41]. Specifically, loss of Wt1 has been reported to induce transdifferentiation of human pleural mesothelial cells into myofibroblastic cells, suggesting that Wt1 is required for the maintenance of mesothelial homeostasis [41]. This is not surprising given that Wt1 has been shown to be involved in the regulation of EMT and MET processes [42]. The downregulation of *Cdh1* and *Krt8* we observed in this study could therefore be linked to the reduction in *Wt1* expression in MCs over passages. Nevertheless, our results suggest that long-term cultured mouse MCs remained in a status whereby epithelial markers are lost only partially since ZO1 was still detectable in a robust punctate pattern around the cell perimeter, and mesenchymal markers were not significantly upregulated.

Because of their ability to proliferate over many passages, we tested the mouse mesothelial cells for clonogenic potential. In contrast to kidney stem cells (KSCs) isolated from mouse newborn kidneys which are clonogenic but give rise to clonal lines with different renal phenotypes [43], mesothelial cells showed properties similar to mesenchymal stem cells (MSCs), since MSCs are able to generate clones with characteristics of the parent cell type only [44].

The capacity to differentiate mesenchymal stem cells towards the mesodermal lineages, specifically adipocytes and osteocytes, has been established as an important parameter of their stemness [45]. This has been exploited in studies to demonstrate that rat and human mesothelial cells have the potential to differentiate towards the adipogenic and osteogenic lineage [5, 45]. Following a similar approach, we could show that upon appropriate stimulation, mouse mesothelial cells adopted phenotypes and expressed genes that are indicative of differentiation steps of adipogenesis and osteogenesis. Our analysis revealed that the differentiation potential for osteogenesis is retained in cells up to passage 13, while cells of higher passage failed to significantly respond. Similarly, MCs of passage 5 could differentiate towards an adipogenic fate, while this differentiation potential was reduced in MCs of higher passages. The decline in the differentiation potential of long-term cultured mesothelial cells could be reflected in the changes in expression levels of some of the stem cell markers analysed. Therefore, our results suggest that the differentiation potential, and in effect stemness of the mesothelial cells, could only be maintained for a limited time under the culture conditions we used.

Since mesothelial cells showed evidence of stemness and differentiation potential, we asked whether they had the potential to respond to a nephrogenic environment by differentiating into kidney cells. However, since mesothelial cells share some characteristics with MSCs, it is possible that they would have a negative effect on *ex vivo* nephron development similarly to MSCs [31]. A recently developed *ex vivo* embryonic kidney rudiment assay lends itself to address these questions since the experimental procedure involves the dissociation of embryonic kidneys into single cells. Exogenous embryonic, adult or stem cells are then mixed in with the embryonic kidney suspension before culture as pelleted chimeric rudiment [33, 46]. Labelling the exogenous cells with lentiviral GFP, Quantum Dots or fluorescent vital dyes allows their identification in the chimeric rudiments, in order to determine whether cell integration into the developing rudiment and furthermore, contribution to nephron structures has taken place. Using this approach, several studies have now demonstrated that stem cells from various sources have the capacity to integrate into chimeric kidney rudiments. In some cases this involves the contribution of exogenous cells to developing glomeruli, comma- and S-shaped bodies [31, 47–49]. Here, we demonstrate that mouse mesothelial cells localise inside the chimeric rudiments, without disrupting the development of the overall kidney rudiments and their structures. This is in clear contrast to mesenchymal stem cells, which, despite expressing some of the key regulators of kidney development, have been shown to disrupt nephron formation in the chimeric rudiments, indicating that not all stem cells have the capacity to support and interact with the developing nephron structures [31, 47].

Within the chimeric rudiments, mesothelial cells were occasionally found in the nephrogenic mesenchyme, where they showed co-expression with the nephron progenitor regulators Pax2 and Wt1 by immunofluorescence. Interestingly, *Pax2* expression could not be detected in the FACS-sorted MCs after co-culture in the chimeric rudiments, suggesting that the number of Pax2+ MCs was very small. We also noted that mesothelial cells aligned robustly with the basement membranes of the forming proximal tubules. Overall, the heterogeneous distribution of MCs surrounding nascent glomeruli and tubular structures was reflected in the up-regulation of a range of mostly mesenchymal or EMT markers in the MCs sorted from the chimeric rudiments after 7 days of culture.

Because the mesothelial cells used for the chimeric kidney rudiment assays had been of higher passages (P22-32) due to the lentvirus transduction protocol employed followed by expansion and FAC sorting of the cells, it is possible that the MC^GFP+^ cells had reached a stage in the long-term culture where their peak differentiation potential had been passed. Therefore, we cannot exclude the possibility that mesothelial cells of earlier passages would have shown a more nephrogenic differentiation response.

We conclude that clonogenicity, stem cell marker expression and differentiation capacity observed in mesothelial cells up to passage 10–13, together provide evidence for stem or progenitor cell characteristics in cultured mouse mesothelial cells. This finding is in contrast to a previous report that mesothelial cells isolated from human pericardial fluid and rat omentum could display stem cell characteristics only up to passage 3 [5].

Our results suggest that during long-time culture, mesothelial cells downregulate specific sets of genes that are part of their gene signature. Importantly, during the early phase of the culture period they exhibit certain characteristics of stemness. However, it is questionable whether the downregulation of gene expression observed during long-term culture can be described as dedifferentiation in the true sense since dedifferentiation is defined as the change of a differentiated cell to a stem or progenitor cell [34]. Thus, further analyses are required to determine the molecular mechanisms that regulate the differences between human and mouse mesothelial cells in culture, and the changes observed during the earlier and later passages. Understanding these mechanisms will allow the development of specific culture conditions and manipulations in order to maintain a status of stem or progenitor characteristics in long-term cultured mesothelial cells, with the view to exploring their potential as regenerative medicine therapies.

## Supporting Information

S1 Fig Mesothelial cells were counted using the trypan blue exclusion assay between passages 5 to 16. Although a spike could be seen at P7, the population doubling time was relatively stable averaging at 25 hours.
(DOCX)

S2 Fig Whole embryonic kidney rudiments (eControl rudiments) from E13.5 mouse embryos were sub-cultured for 7 days on air-to-media interface. The *ex vivo* culture conditions did not affect the development of nephron structures in eControl rudiments, as shown by immuno-labelling for Six2, Wt1 and Pax2 (A, D, G). Similarly, proximal and distal tubule structures developed in the eControl rudiment culture which were detected through megalin (B) and PNA lectin (E, H) staining respectively. Scale bars are 50 μM (A-F, G-I).
(DOCX)

S3 Fig E13.5 re-aggregated kidneys rudiments (rControl) formed nephron structures. Immunostaining for Wt1 and laminin indicated the presence of developing nephrons, including nascent glomeruli after 7 days of culture (A-F). Immunolabelling for megalin and laminin at E13.5+7 showed the presence of extensive proximal tubules across the rudiments (G-I). Scale bars are 100 μM (A-C) and 50 μM (D-F, G-I).
(DOCX)

S4 Fig Typical examples of reaggregated chimeric kidney rudiments containing MC^GFP+^ cells at a ratio of 1:10. (A) Chimeric rudiment at day 1. (B) Chimeric rudiment at day 4. Scale bar 200 μm (A) and 100 μm (B).
(DOCX)

S5 Fig A cluster heat map denoting fold changes (over normalized means) for a number of biomarkers in passaged mesothelial cells (P5-P25) and the omentum culture explants (control). The gene expression values plotted were averages generated from 3 biological replicas. Gene upregulation is represented in red, downregulation is green, and no changes in relative expression is black; as generated using the GENE-E software.
(DOCX)

S1 Table List of primers for qPCR analysis. (DOCX)

S2 Table qPCR results as dCt and fold change (RQ), including statistical analysis. One way ANOVA was used to compare and calculate statistical significance of all samples, and Tukey’s post-hoc revealed significance in the comparison of individual samples with OMC: **** = P<0.0001, *** = P<0.001, ** = P<0.01 and * = P<0.05.
(DOCX)
