# Supplementary material for: Characterisation of Cultured Mesothelial Cells Derived from the Murine Adult Omentum
Source: PLoS One. 2016 Jul 12;11(7):e0158997. doi: 10.1371/journal.pone.0158997 (PMC4942062; doi:10.1371/journal.pone.0158997)
Supplement: S1 Fig — Although a spike could be seen at P7, the population doubling time was relatively stable averaging at 25 hours. (DOCX) [file pone.0158997.s001.docx]

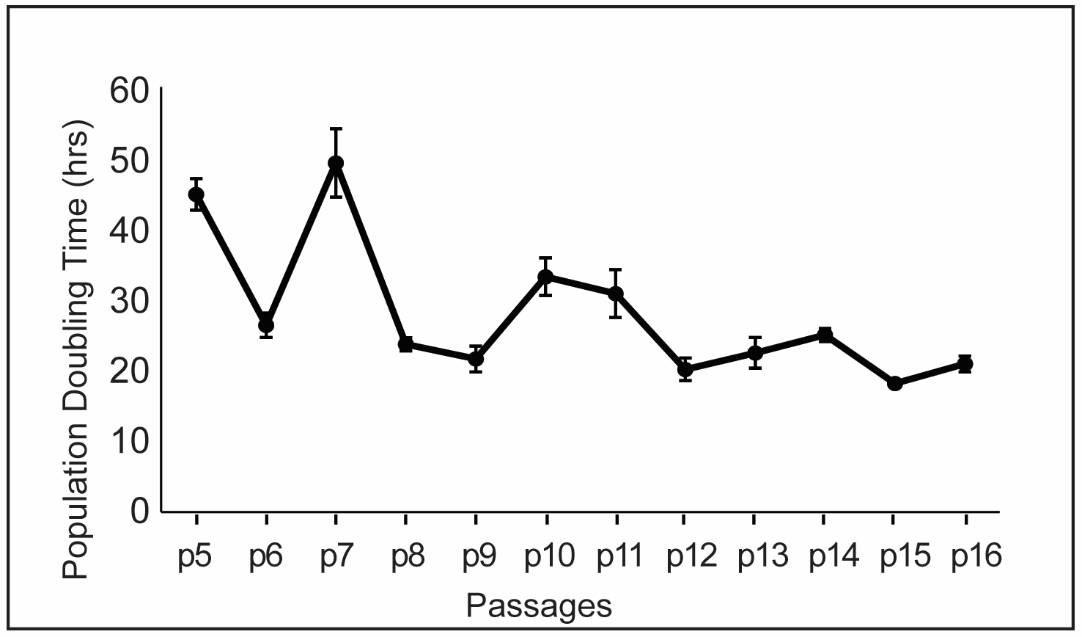


**Figure S1.** Mesothelial cells were counted using the trypan blue exclusion assay between passages 5 to 16. Although a spike could be seen at P7, the population doubling time was relatively stable averaging at 25 hours.
