# Supplementary material for: Characterisation of Cultured Mesothelial Cells Derived from the Murine Adult Omentum
Source: PLoS One. 2016 Jul 12;11(7):e0158997. doi: 10.1371/journal.pone.0158997 (PMC4942062; doi:10.1371/journal.pone.0158997)
Supplement: S2 Fig — The ex vivo culture conditions did not affect the development of nephron structures in eControl rudiments, as shown by immuno-labelling for Six2, Wt1 and Pax2 (A, D, G). Similarly, proximal and distal tubule structures developed in the eControl rudiment culture which were detected through megalin (B) and PNA lectin (E, H) staining respectively. Scale bars are 50 μM (A-F, G-I). (DOCX) [file pone.0158997.s002.docx]

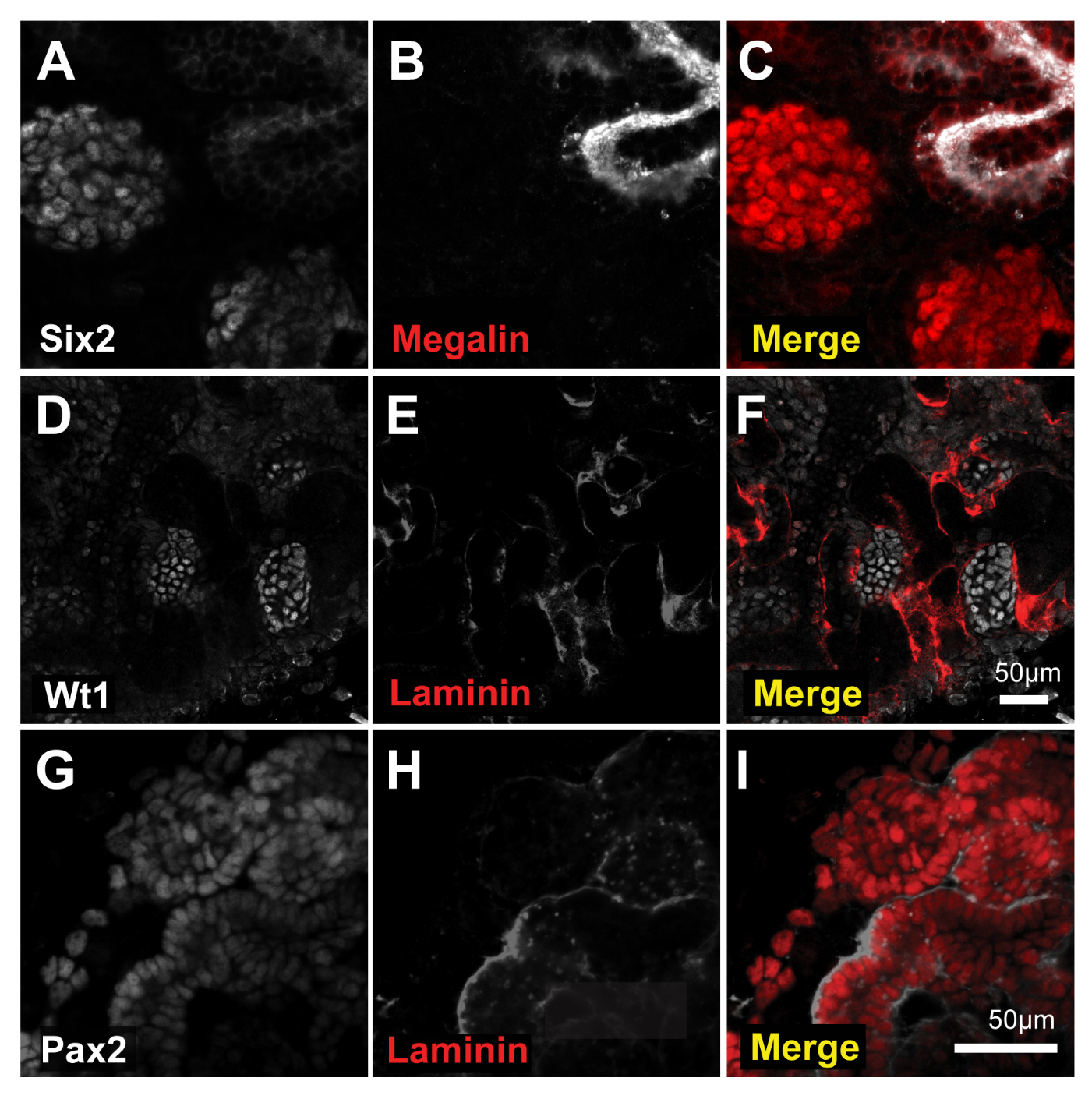


**Figure S2.** Whole embryonic kidney rudiments (eControl rudiments) from E13.5 mouse embryos were sub-cultured for 7 days on air-to-media interface. The *ex vivo* culture conditions did not affect the development of nephron structures in eControl rudiments, as shown by immuno-labelling for Six2, Wt1 and Pax2 (A, D, G). Similarly, proximal and distal tubule structures developed in the eControl rudiment culture which were detected through megalin (B) and PNA lectin (E, H) staining respectively. Scale bars are 50 µM (A-F, G-I).
