# Supplementary material for: Characterisation of Cultured Mesothelial Cells Derived from the Murine Adult Omentum
Source: PLoS One. 2016 Jul 12;11(7):e0158997. doi: 10.1371/journal.pone.0158997 (PMC4942062; doi:10.1371/journal.pone.0158997)
Supplement: S3 Fig — Immunostaining for Wt1 and laminin indicated the presence of developing nephrons, including nascent glomeruli after 7 days of culture (A-F). Immunolabelling for megalin and laminin at E13.5+7 showed the presence of extensive proximal tubules across the rudiments (G-I). Scale bars are 100 μM (A-C) and 50 μM (D-F, G-I). (DOCX) [file pone.0158997.s003.docx]

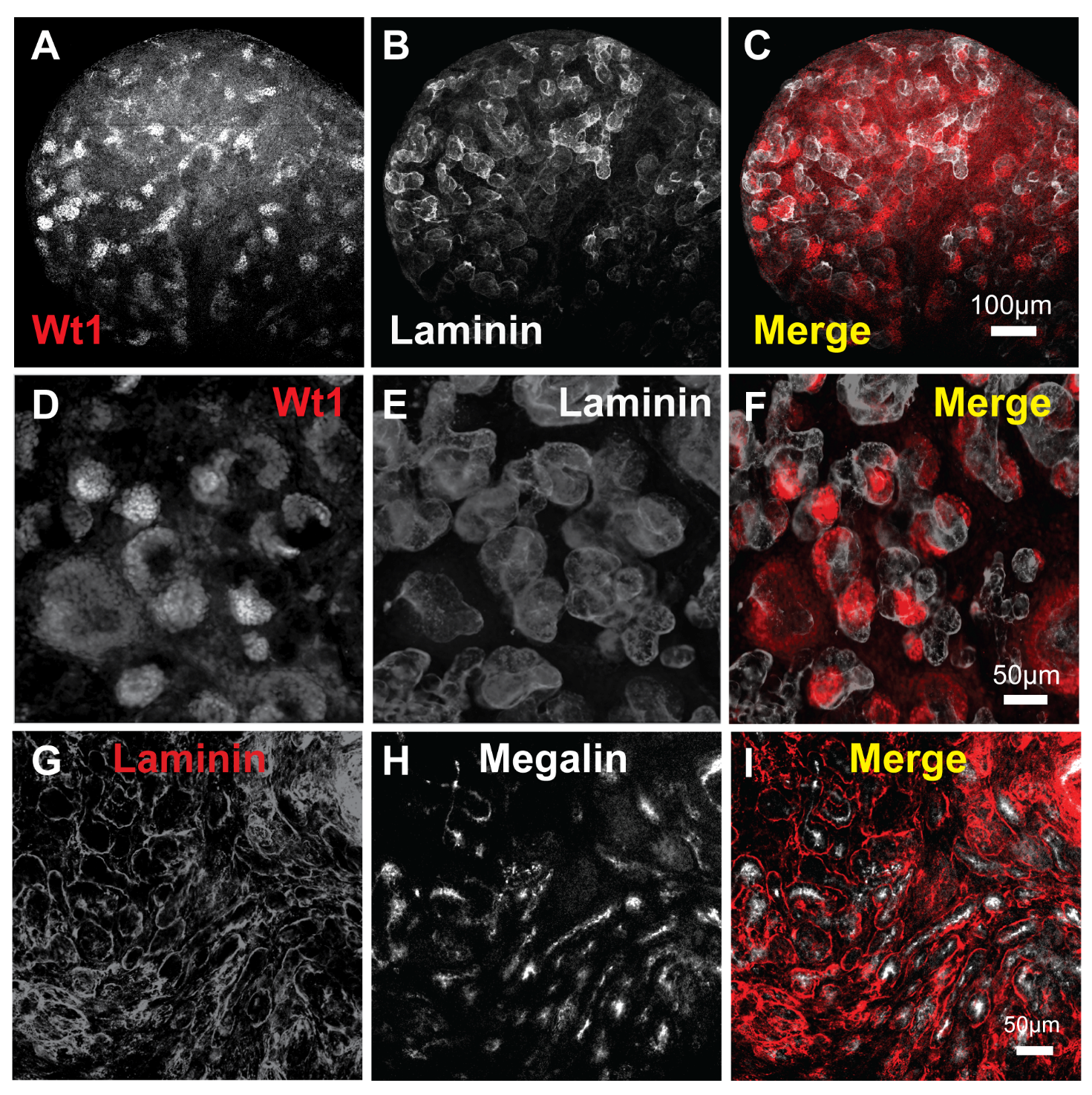


**Figure S3**. E13.5 re-aggregated kidneys rudiments (rControl) formed nephron structures. Immunostaining for Wt1 and laminin indicated the presence of developing nephrons, including nascent glomeruli after 7 days of culture (A-F). Immunolabelling for megalin and laminin at E13.5+7 showed the presence of extensive proximal tubules across the rudiments (G-I). Scale bars are 100 µM (A-C) and 50 µM (D-F, G-I).
