# Supplementary material for: Characterisation of Cultured Mesothelial Cells Derived from the Murine Adult Omentum
Source: PLoS One. 2016 Jul 12;11(7):e0158997. doi: 10.1371/journal.pone.0158997 (PMC4942062; doi:10.1371/journal.pone.0158997)
Supplement: S4 Fig — (A) Chimeric rudiment at day 1. (B) Chimeric rudiment at day 4. Scale bar 200 μm (A) and 100 μm (B). (DOCX) [file pone.0158997.s004.docx]

**
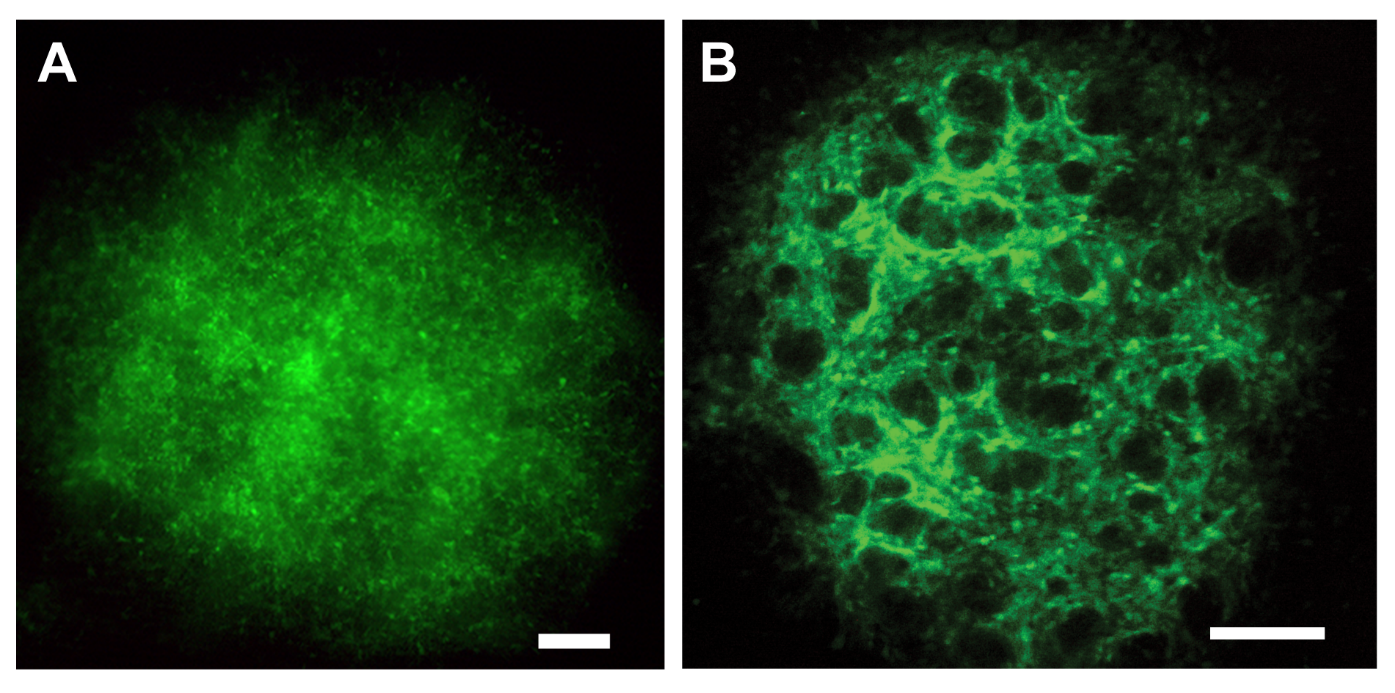
**

**Figure S4.** Typical examples of reaggregated chimeric kidney rudiments containing MC^GFP+^ cells at a ratio of 1:10. (A) Chimeric rudiment at day 1. (B) Chimeric rudiment at day 4. Scale bar 200 μm (A) and 100 μm (B).
