# Supplementary material for: Characterisation of Cultured Mesothelial Cells Derived from the Murine Adult Omentum
Source: PLoS One. 2016 Jul 12;11(7):e0158997. doi: 10.1371/journal.pone.0158997 (PMC4942062; doi:10.1371/journal.pone.0158997)
Supplement: S5 Fig — The gene expression values plotted were averages generated from 3 biological replicas. Gene upregulation is represented in red, downregulation is green, and no changes in relative expression is black; as generated using the GENE-E software. (DOCX) [file pone.0158997.s005.docx]

**
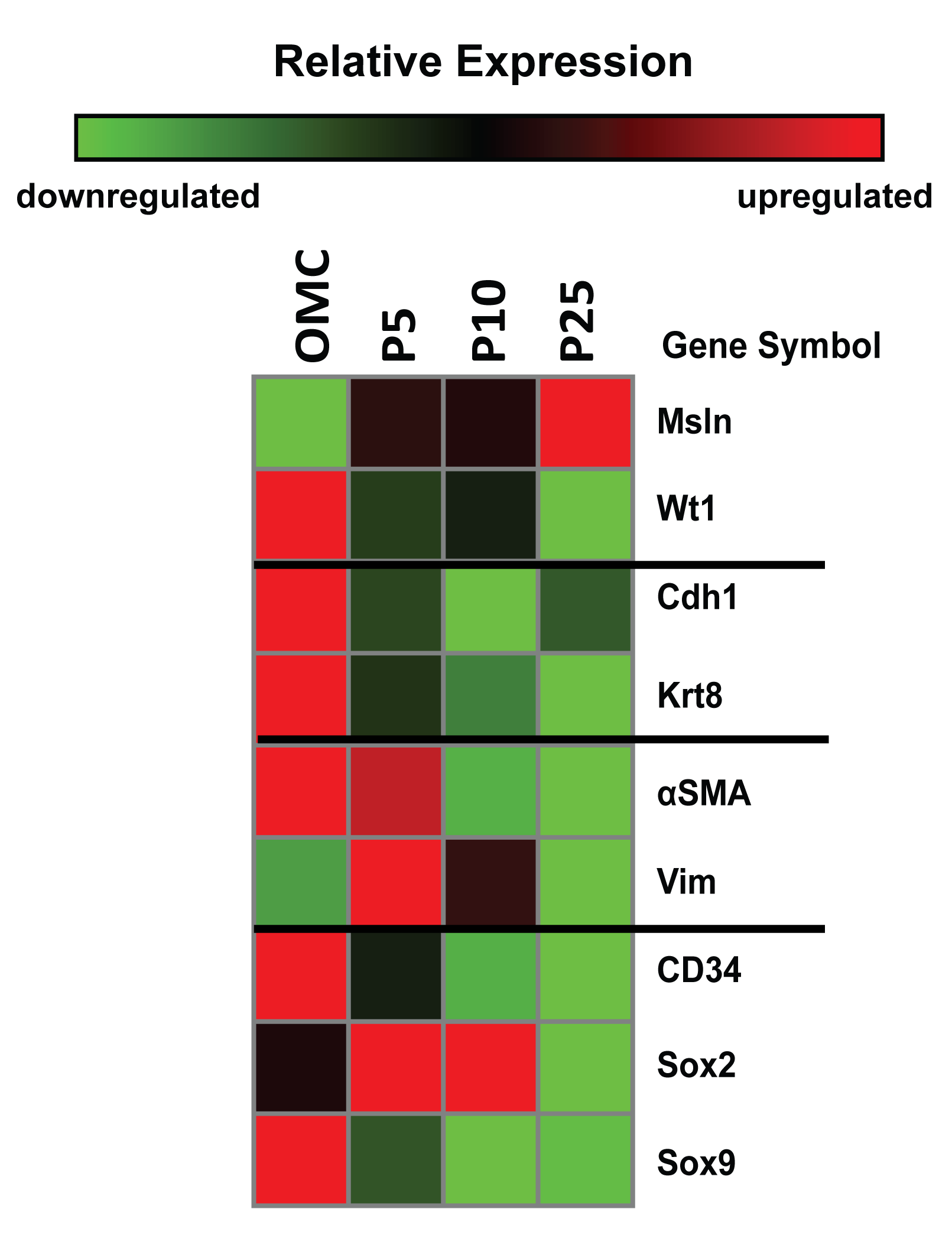
**

**Figure S5.** A cluster heat map denoting fold changes (over normalized means) for a number of biomarkers in passaged mesothelial cells (P5-P25) and the omentum culture explants (control). The gene expression values plotted were averages generated from 3 biological replicas. Gene upregulation is represented in red, downregulation is green, and no changes in relative expression is black; as generated using the GENE-E software.
