# Supplementary material for: Characterisation of Cultured Mesothelial Cells Derived from the Murine Adult Omentum
Source: PLoS One. 2016 Jul 12;11(7):e0158997. doi: 10.1371/journal.pone.0158997 (PMC4942062; doi:10.1371/journal.pone.0158997)
Supplement: S1 Table — (DOCX) [file pone.0158997.s006.docx]

**Table S1.**

| Gene | Forward primers | Reverse primers | Melting temperature | Product length |
| --- | --- | --- | --- | --- |
| Gapdh | CATCTTCCAGGAGCGAGACC | CTGGAAGGTGGACAGTGAGG | 60°C/ 62°C | 150 |
| β-actin | GTACCCAGGCATTGCTGACA | CTGGAAGGTGGACAGTGAGG | 60°C/ 62°C | 145 |
| Wt1 | AATGCGCCCTACCTGCCCA | CCGTCGAAAGTGACCGTGCTGTAT | 60°C/ 62°C | 116 |
| Msln | ATGTACTCCCACGGAGGTCT | GCCACAAATTTCCCAGGCAG | 60°C | 145 |
| Krt8 | GATGAACCGCAACATCAACCG | GGTCTGGGCATCCTTAATGGC | 60°C | 127 |
| Chd1 | GCTCTCATCATCGCCACAGA | GCAGTAAAGGGGGACGTGTT | 60°C | 197 |
| ZO1 | TGCCATTACACGGTCCTCTG | AGGGACTGGAGATGAGGCTT | 60°C | 175 |
| Vim | TCTGCCTCTGCCAACCTTTT | ACCTGTCCATCTCTGGTCTCA | 60°C | 126 |
| αSMA | AGAGGCACCACTGAACCCTA | CACCATCTCCAGAGTCCAGC | 60°C | 158 |
| Sox9 | CAAGACTCTGGGCAAGCTCT | CCGGGGCTGGTACTTGTAATC | 60°C | 124 |
| Sox2 | CACATGTGAGGGCTGGACTG | TCCTCTTTTTGCACCCCTCC | 60°C | 146 |
| CD34 | TCATCTTCTGCTCCGAGTGC | GCCTCAGCCTCCTCCTTTTC | 60°C | 176 |
| Pparγ | ATTGAGTGCCGAGTCTGTGG | GCAAGGCACTTCTGAAACCG | 60°C | 191 |
| Sparc | TGTTGGCCCGAGACTTTGAG | CGTGTGGTGCAATGTTCCAT | 60°C | 160 |
| Sdf1 | GCTCTGCATCAGTGACGGTA | TCAGATGCTTGACGTTGGCT | 62°C | 98 |
| Snail1 | AGCCCAACTATAGCGAGCTG | GTAGGGCTGCTGGAAGGTGA | 60°C | 112 |
| Snail2 | CGAACCCACACATTGCCTTG | GTGAGGGCAAGAGAAAGGCT | 60°C/ 62°C | 117 |
| Zeb1 | GGGGAAACCGCAAGTTCAAG | AGCCAGAATGGGAAAACCGT | 60°C | 141 |
| Twist1^$^ | AGCGGGTCATGGCTAACG | GGACCTGGTACAGGAAGTCGA | 60°C | 162 |

^$^ (Xiang et al., 2011)

Table S1. List of primers for qPCR analysis.

References:

XIANG, X., ZHUANG, X., JU, S., ZHANG, S., JIANG, H., MU, J., ZHANG, L., MILLER, D., GRIZZLE, W. & ZHANG, H. G. 2011. miR-155 promotes macroscopic tumor formation yet inhibits tumor dissemination from mammary fat pads to the lung by preventing EMT. *Oncogene,* 30**,** 3440-3453.
