# Supplementary material for: Characterisation of Cultured Mesothelial Cells Derived from the Murine Adult Omentum
Source: PLoS One. 2016 Jul 12;11(7):e0158997. doi: 10.1371/journal.pone.0158997 (PMC4942062; doi:10.1371/journal.pone.0158997)
Supplement: S2 Table — One way ANOVA was used to compare and calculate statistical significance of all samples, and Tukey’s post-hoc revealed significance in the comparison of individual samples with OMC: **** = P<0.0001, *** = P<0.001, ** = P<0.01 and * = P<0.05. (DOCX) [file pone.0158997.s007.docx]

**Table S2.**

| **Sample** | **dCt** | **SEM** | **Fold Change** | **SEM** | **One way ANOVA** |
| --- | --- | --- | --- | --- | --- |
| *Wt1* | | | | | P<0.0001 |
| OMC | 6.50 | 0.09 | 1.00 | 0.06 |  |
| P5 | 18.56 | 0.44 | 2.34E-4 | 6.18E-5 | **** |
| P10 | 17.01 | 0.80 | 6.88E-4 | 2.94E-4 | *** |
| P25 | 25.22 | 3.41 | 2.32E-06 | 2.10E-6 | **** |
| *Msln* | | | | | P<0.0001 |
| OMC | 11.38 | 0.29 | 1.00 | 0.18 |  |
| P5 | 9.02 | 0.50 | 5.13 | 1.51 |  |
| P10 | 9.11 | 0.58 | 4.82 | 1.59 | *** |
| P25 | 7.40 | 0.16 | 15.69 | 1.66 | ** |
| *Krt8* | | | | | P=0.0007 |
| OMC | 11.30 | 0.04 | 1.00 | 0.03 |  |
| P5 | 16.68 | 1.30 | 0.02 | 0.01 | *** |
| P10 | 18.27 | 0.45 | 7.97E-3 | 2.13E-3 | ** |
| P25 | 19.96 | 0.79 | 2.48E3 | 1.04E-3 | ** |
| *Cdh1* | | | | | P=0.0137 |
| OMC | 12.85 | 0.1 | 1.00 | 0.07 |  |
| P5 | 20.63 | 3.83 | 4.53E-3 | 4.21E-3 |  |
| P10 | 24.59 | 4.09 | 2.91E-4 | 2.74E-4 | * |
| P25 | 21.20 | 0.91 | 3.05E-3 | 1.43E-3 | * |
| *Vim* | | | | |  |
| OMC | 3.45 | 0.35 | 1.00 | 0.22 |  |
| P5 | 2.34 | 0.15 | 2.16 | 0.21 |  |
| P10 | 2.83 | 0.37 | 1.53 | 0.35 |  |
| P25 | 3.61 | 0.249 | 0.90 | 0.14 |  |
| *αSMA* | | | | |  |
| OMC | 2.44 | 0.06 | 1 | 0.04 |  |
| P5 | 2.96 | 0.29 | 0.70 | 0.13 |  |
| P10 | 6.42 | 0.48 | 0.06 | 0.02 |  |
| P25 | 6.76 | 2.93 | 0.05 | 0.04 |  |

| *Sox2* | | | | |  |
| --- | --- | --- | --- | --- | --- |
| OMC | 15.18 | 0.19 | 1.00 | 0.12 |  |
| P5 | 14.42 | 0.68 | 1.69 | 0.64 |  |
| P10 | 14.40 | 0.187 | 1.71 | 0.21 |  |
| P25 | 16.16 | 0.35 | 0.51 | 0.11 |  |
| *Sox9* | | | | | P=0.0089 |
| OMC | 8.77 | 0.49 | 1.00 | 0.29 |  |
| P5 | 11.27 | 0.55 | 0.18 | 0.06 | * |
| P10 | 12.33 | 1.47 | 0.08 | 0.05 |  |
| P25 | 12.25 | 0.24 | 0.09 | 0.01 | ** |
| *CD34* | | | | | P=0.0053 |
| OMC | 7.25 | 1.48 | 1.00 | 0.64 |  |
| P5 | 12.18 | 1.25 | 0.03 | 0.02 | * |
| P10 | 15.29 | 1.16 | 3.80E-3 | 2.10E-3 | ** |
| P25 | 15.99 | 1.49 | 2.35E-3 | 1.51E-3 | ** |

Table S2. qPCR results as dCt and fold change (RQ), including statistical analysis. One way ANOVA was used to compare and calculate statistical significance of all samples, and Tukey’s post-hoc revealed significance in the comparison of individual samples with OMC: ****=P<0.0001, ***=P<0.001, **=P<0.01 and *=P<0.05.
